# Development of a Serial Order in Speech Constrained by Articulatory Coordination

**DOI:** 10.1371/journal.pone.0078600

**Published:** 2013-11-05

**Authors:** Hiroki Oohashi, Hama Watanabe, Gentaro Taga

**Affiliations:** 1 Graduate School of Education, The University of Tokyo, Tokyo, Japan; 2 Research Fellow of the Japan Society for the Promotion of Science, Koujimachi Business Center, Tokyo, Japan; Northwestern University, United States of America

## Abstract

Universal linguistic constraints seem to govern the organization of sound sequences in words. However, our understanding of the origin and development of these constraints is incomplete. One possibility is that the development of neuromuscular control of articulators acts as a constraint for the emergence of sequences in words. Repetitions of the same consonant observed in early infancy and an increase in variation of consonantal sequences over months of age have been interpreted as a consequence of the development of neuromuscular control. Yet, it is not clear how sequential coordination of articulators such as lips, tongue apex and tongue dorsum constrains sequences of labial, coronal and dorsal consonants in words over the course of development. We examined longitudinal development of consonant-vowel-consonant(-vowel) sequences produced by Japanese children between 7 and 60 months of age. The sequences were classified according to places of articulation for corresponding consonants. The analyses of individual and group data show that infants prefer repetitive and fronting articulations, as shown in previous studies. Furthermore, we reveal that serial order of different places of articulations within the same organ appears earlier and then gradually develops, whereas serial order of different articulatory organs appears later and then rapidly develops. In the same way, we also analyzed the sequences produced by English children and obtained similar developmental trends. These results suggest that the development of intra- and inter-articulator coordination constrains the acquisition of serial orders in speech with the complexity that characterizes adult language.

## Introduction

Speech production is very complex; the central nervous system coordinates more than 100 muscles to control movements of jaw and soft tissues such as the lips and tongue [1], [2]. This property enables us to perform an extraordinarily rapid sequence of action, that is, the production of dozens of syllables or phonemes in a single breath. This phenomenon is related to the serial order problem [3], which addresses how behaviors are sequenced without triggers from sensory feedback. The serial order in speech production has been a central issue in the phonological study that attempts to find universal structures that govern all human languages [4]. Other lines of study focusing on motor control of articulatory systems have argued that discrete phonemic units in human languages are founded on the dynamical nature of the neuromuscular system that controls precise coordination among many articulators [5]–[9]. Moreover, studies on the acquisition of speech production have attempted to interpret phonological structures preferred in language and babbling in terms of the articulatory system [10]–[13]. Aside from neuromuscular coordination, vocal tract anatomy in early development differs from those of adults in that infants’ vocal tracts are not only smaller than adults’, but they have a broader oral cavity, a tongue mass that is proportionally larger and more anterior, and a more gradually sloping pharyngeal tract [14]. Findings from these studies suggest that a neuromuscular coordination and the vocal tract anatomy constrain the development of phonotactics for words in a language.

**Figure 1:**
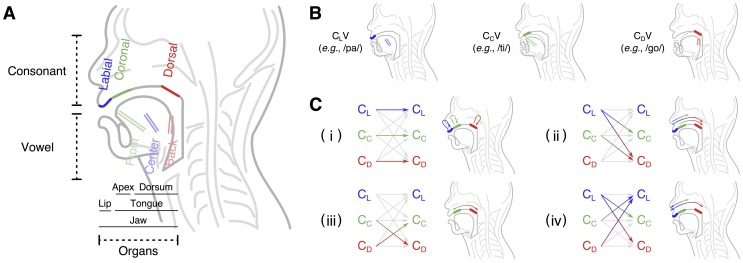
The classification of serial order in articulations in the previous and present studies. (A) The place of articulations for consonants and vowels, and articulatory organs involved in each consonant. Depending on the horizontal position of the tongue, vowels are categorized into three types including front, center and back. This figure illustrates three places of articulations, including labial, coronal and dorsal. Labial consonants are mainly articulated by the lips and jaw. Coronal consonants are mainly articulated by the tongue apex and jaw. Dorsal consonants are mainly articulated by the tongue dorsum and jaw. (B) Three consonant-vowel patterns preferred by infants in early development. Focusing on three consonantal and vowel categories, theoretically speaking, it is possible to produce nine consonant-vowel sequences. However, infants prefer three out of those nine possible sequences: labial-center, coronal-front, and dorsal-back [11], [12]. (C) Serial order in articulation of consonants in consonant-vowel-consonant(-vowel) sequences. In the present study, focusing on the relationship among articulators producing adjacent consonants, we divided sequences into four categories: (i) Sequences consists of consonants produced at the same place of articulation. (ii) Sequences produced by movements from more anterior place to posterior one. (iii) Sequences consist of coronal and dorsal consonants, which are articulated by the same organ but different places (intra-organ articulations). (iv) Sequences consist of labial and coronal/dorsal consonants, which are articulated by different organs: lips and tongue (inter-organ articulations).

The human articulatory system consists of organs, such as the jaw, tongue and lips (Figure 1A). In order to produce speech sounds, speakers coordinate these organs to change the vocal tract configuration. In brief, these articulatory movements are close-open alternations of the vocal tract that generate series of consonant and vowels.

Vowels are mainly characterized by the horizontal and vertical positions of the tongue. According to the horizontal positions of the tongue, vowels are classified into front (e.g., [i] end [e]), center (e.g., [a]) and back vowels (e.g., [u] and [o]). According to the vertical positions of the tongue, vowels are classified into high (e.g., [i] and [u]), middle (e.g., [e] and [o]) and lower vowels (e.g., [a]). Consonants can be classified into categories according to their places of articulation. Of these, labial (e.g., [p] and [m]), coronal (e.g., [t] and [d]) and dorsal consonants (e.g., [k] and [g]) are the major categories. Consonants are also classified by the manner of articulation, such as fricative (e.g., [s] and [z]), nasal (e.g., [m] and [n]), and stop (e.g., [p] and [t]). Consonants are produced as a result of the coordinated movements of one or more articulators; these movements are referred to as consonantal gestures. The lips, tongue apex and tongue dorsum articulate to produce labial, coronal and dorsal consonants, and the jaw can contribute to all of these consonants. Both coronal and dorsal consonants are produced by the tongue but they are governed by different tongue tissues. Regarding the development of speech sounds, the previous study [12] has shown that only a small set of sounds are used frequently in babbling. Stop and nasal consonants with labial, coronal and dorsal places of articulation and lower-front and lower-center vowels are present in infants from all language environments from the beginning of the babbling stage and remain in place in the first-word stage.

As children acquire speech production, previous analyses have revealed a shared preference for specific articulatory patterns [11], [12]. For example, specific consonant-vowel (consonants and vowels are hereafter described as C and V, respectively) patterns are observed at significantly high frequencies in the period of babbling (Figure 1B). Regarding the order of consonant in a CVC(V) sequence, the same consonantal gestures are repeated (Figure 1C-i). Studies have also shown that both children and adults produce labial-vowel-coronal sequences more frequently than coronal-vowel-labial [11], [12]. A previous study [15] showed more generally that the first consonant in words produced by children aged between 16 and 24 months has a more anterior place of articulation than the second one (Figure 1C-ii), and this pattern has been referred to as fronting. These patterns in child speech production are observed not only in many modern languages but also in proto-language corpora [11], [12]. Note that there is no preference for the sequences in adult Japanese [16], [17].

For speech production, one articulator must coordinate with other articulators and this coordination can vary from phoneme to phoneme. Therefore, not only simultaneous coordination of articulatory organs but also their sequential coordination is critical for a serial order in speech production. Focusing on neuromuscular coordination, previous studies emphasize the important roles of the jaw in development of speech production [11], [12]. The preferred CV patterns and repetitions of CVC(V) sequences in babbling are generated by rhythmic jaw cycles on which speech production is founded. According to this explanation, a resting position of the tongue and an inertia caused by close-open alternation of the jaw generate the CV patterns, such as/papa/,/dede/and/gogo/. Among the CV patterns preferred in babbling, these studies [11], [12] especially focus on sequences consisting of labial consonants and center vowels supposing that mere jaw raising gestures would induce a labial closure of the vocal tract. Other studies [18] also show that the mandibular oscillations with passive tongue configurations could induce a coronal closure of the vocal tract. Furthermore it has been speculated that the mandibular oscillations can be controlled by the central pattern generators, which may work in the brain stem of infants from early on [12], [19]. As development proceeds, independent and active recruitment of the different part of the tongue and the lips into the cycle causes increases in the variety of lingual consonants used in speech production. Regarding this divergent process, the previous studies mainly focus on the ordering of labial and coronal consonants [11], [12] (Figure 1C-ii), which is produced on the basis of the inter-articulatory coordination between the lips and tongue apex. Previous kinematic studies [20]–[22] also show that development of the coordinative organization of the jaw and lips during speech production for a specific sound can be characterized by distinct phases, in which movements of the jaw become differentiated and integrated with movements of the lips.

In addition to the repetition and fronting that dominate early speech, the present study examines the longitudinal development of CVC(V) sequences produced by serial coordination of articulators such as the lip, tongue apex and tongue dorsum. In particular, we focus on an intra-articulator and an inter-articulator serial order (Figures 1C-iii and iv). An intra-articulator serial coordination between the tongue apex and dorsum (Figure 1C-iii) might be constrained by a physical connection within the tongue tissue and/or by a limited neural control of articulators in the period of babbling, and are established through the later development of the neuromuscular system. An inter-articulator serial coordination between the lips and tongue dorsum as well as between the lips and tongue apex (Figure 1C-iv) might be also constrained by a developing mechanism for the neuromuscular control of these articulators.

If the intrinsic property of the neuromuscular system constrains early developments of speech production, language universal developmental patterns could be observed. On the other hand, lexical specifications and linguistic inputs facilitate infants’ speech production of their native language [23], [24]. In order to distinguish between neuromuscular maturation and linguistic stimulations on development of speech production, cross-linguistic comparison between different languages and analysis of child-directed speech are required.

To that end, we used a Japanese corpus, which aimed to longitudinally track early normal speech development; children and their parents were not required to do any particular task other than to record their talk during everyday situations in daily life [25]. The database has been used to reveal longitudinal changes in fundamental frequency, formant frequencies and conversational style [26]–[28]. Thus, we further studied CVC(V) sequences reflecting the coordination of articulators. In the same way, we performed analysis using an English database [29], which is provided in the CHILDES projects [30]. This database also aimed to record infants’ naturally occurring vocalizations in their home environment, usually in the company of a parent and/or siblings [31]. Through data collections for this database, the previous studies have revealed that some CV patterns and labial-coronal fronting patterns are preferred in early development [11], [12]. In the present study, we investigate longitudinal changes in CVC(V) productions in Japanese and English to examine whether serial coordination of articulators acts as a constraint for the developmental emergence of sequences in words.

## Methods

Data for this study are taken from the NTT Japanese infant speech database [25] and the Davis corpus for English [29], [30]. The Japanese corpus longitudinally tracks five children from birth to 60 months old. The English corpus contains data of 21 children and tracking periods of it ranges from five to 27 months. For both databases, well-trained transcribers listened to the speech files and transcribed utterances, and checked reliability (for details see [25], [31]). The utterances were segmented from continuous speech based on the pause duration. In the present study, we used transcriptions of utterances by three children aged 7 to 60 months (Table S1), transcriptions of child-directed speech by their parents (Table S2), and duration data for each phoneme for Japanese. Because of lacks of the transcriptions, we did not use data of the other two children contained in the Japanese corpus. We also used transcription of utterances by 21 children aged 7 to 37 months for English (Table S3). From the transcriptions, we extracted CVC units contained in sequences such as CVC and CVCV, and examined changes in place of articulation patterns through stages of development. For instance, in the case of C_1_VC_2_VC_3_, we extracted two sequences C_1_VC_2_ and C_2_VC_3_. In this study, we focused on only three types of consonants: labial stop and nasal consonants ([p], [b] and [m], hereafter C_L_), coronal stops and nasals ([t], [d] and [n], hereafter C_C_) and dorsal stops ([k] and [g], hereafter C_D_), which are favored from the beginning of the babbling stage [12]. Therefore, CVC sequences were classified into nine categories; C_L_VC_L_, C_L_VC_C_, C_L_VC_D_, C_C_VC_L_, C_C_VC_C_, C_C_VC_D_, C_D_VC_L_, C_D_VC_C_ and C_D_VC_D_. Consonants and vowels have different characteristics: consonant production has a discrete character generated by transient coordination of articulators and vowels have a relatively continuous character primarily controlled by position of the tongue dorsum. Given this difference, we assumed that the CC relationship in CVC(V) sequences may primarily exhibit combinatory complexity as a source of linguistic complexity. Therefore, we omitted vowels from the present analysis and focused only on the CC sequences. During the extraction process, syllable positions in an utterance were not taken into account, and syllable boundaries and diacritic marks were ignored. All CVC sequences were analyzed to determine the token frequencies of serial order relationship.

### Analysis of individual data for Japanese

For the Japanese analysis, taking into account that the corpus tracks a small number of subjects over an extensively longitudinal period [25], we first conducted individual analysis. We calculated ratios of each occurrence of the nine CVC categories to the total CVC occurrences for each child for each month of age. To analyze serial order in places of articulation for consonants, we calculated four types of developmental curves (Figure 1C): (i) repetitive articulation at the same place of an organ (C_L_VC_L_ + C_C_VC_C_ + C_D_VC_D_), (ii) articulatory movements from more anterior places (C_L_VC_C_ + C_L_VC_D_ + C_C_VC_D_) to posterior ones (C_C_VC_L_ + C_D_VC_L_ + C_D_VC_C_), (iii) different places of articulations within the same organ (C_C_VC_D_ + C_D_VC_C_) and (iv) articulations by different organs (Figure1C-i to -iv). Traditionally, linguistics treat the tongue apex and the tongue dorsum differently (the tongue apex and dorsum are used to articulate coronal consonants and dorsal consonants, respectively). Thus, in terms of (iv), we calculated two values, that is, C_L_VC_C_ + C_C_VC_L_ and C_L_VC_D_ + C_D_VC_L_. In the following, we referred to (i) – (iv) as the repetitions, fronting, intra- and inter-organ articulations.

For (i), we defined and normalized a developmental curve. In detail, we defined the developmental curves by using kernel regression (see Supporting Information) [32]. Although there are many options of curve fitting such as polynomial function, we chose the kernel regression to obtain higher determination coefficients (R^2^) for fitting developmental trends. First, we calculated the Gramian matrix by using the radial basic function as the kernel function. The parameter of the kernel function was decided by the leave-one-out cross-validation method. Next, we set random values into hyper-parameters, deciding on a variance of the weight coefficients and the noise. Then, we calculated means and variances of the Gaussian distribution for the predictor variables and the weight coefficients. Subsequently, we updated the hyper-parameters. Finally, we estimated the optimal predictor variables by the iteration of these processes. In order to normalize an argument trajectory, we divided the trajectory by a maximal value. The goodness-of-fit of the kernel regression for the developmental curve was assessed by the R^2^.

For (ii), we defined developmental curves as follows. When we focus on directions of articulations, the nine CVC categories are also classified into two additional categories: (a) direction of articulations from front to back (C_L_VC_C_ + C_L_VC_D_ + C_C_VC_D_) and (b) direction of articulations from back to front (C_C_VC_L_ + C_D_VC_L_ + C_D_VC_C_). We calculated [(a) – (b)]/[(a) + (b)] as the fronting index and defined a developmental curve in the same manner as (i). When a value of the index is larger than 0, children prefer fronting patterns to backing ones. For (iii) and (iv), we defined developmental curves in the same manner as (i), and normalized them.

Moreover, in order to examine the developmental changes in speed of CVCV production, we also calculated mean durations of CVCV sequences for each child for each month of age, and then defined the developmental curves for the mean durations. To clearly distinguish a unit for analysis, we focused only on CVCV rather than both CVC and CVCV.

### Analysis of group data for Japanese

To assess developmental trends of group data for Japanese, we pooled data across three children and obtained developmental curves (i) – (iv) (Figure 1C) in the same manner as was used for the analysis of individual data. We also obtained a developmental curve of speech rates for the group data. In addition to the analysis of children, we examined if ratios of four types of CVC patterns of child-directed speech by mothers change along with those of their children in the group data of Japanese.

### Analysis of individual data for English

For the English analysis, we first analyzed the individual data of English in the same manner as was done for the analysis of individual data for Japanese. Since the age of sampling and the number of data vary from child to child, we selected two children whose data cover a wide range of ages and contain a large number of samples (hereafter, we refer them as child1 and 2, respectively).

### Analysis of group data for English

To assess developmental trends of group data for English, we also analyzed pooled data across 21 children in the same manner as was done for the analysis of group data for Japanese.

## Results

### Analysis of individual data for Japanese

**Figure 2:**
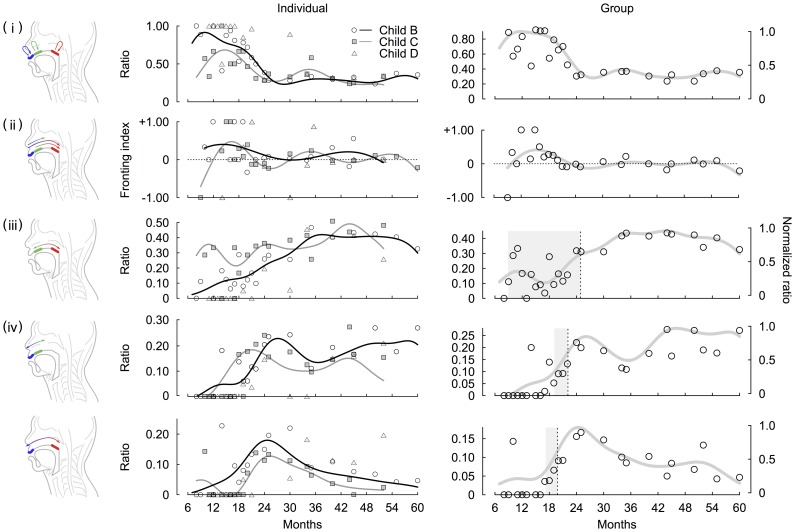
The developmental changes in serial order in articulation of consonants obtained by the analysis of individual and group data for Japanese. Middle and right columns show developmental curves obtained by the analysis of individual and group data for Japanese, respectively. Each row indicates (i) repetitions, (ii) fronting, (ii) intra-organ articulations, and (iv) inter-organ articulations. In the middle column, circles, squares and triangles denote relative ratio of each type of CVC patterns produced by child B, child C and child D, respectively. Black, gray and silver curves indicate that developmental curves of child B, child C and child D, respectively. In the right column, circles and lines denotes relative ratio of each type of CVC patterns obtained from pooled data and developmental curves of them, respectively. The shaded areas indicate periods between onset and offset of the developmental changes. We defined the onsets and offsets as months at which a value of curves exceeded 1/3 and 2/3, respectively.

We conducted individual analysis of two of the three Japanese children. Data of the other one child were not used for the curve fitting because of sparse nature of data sampling. Both children showed early predominance and a later decrease in the case of repetitions (the goodness-of-fit of the kernel regression were R^2^ = 0.765 and 0.573 for child B and C, respectively). Almost all the CVCs were generated by the same place of articulation until around 12 months for child B and 18 months for child C after which the ratio gradually decreased until about 24 months and then it remained the stable (Figure 2i).

**Figure 3:**
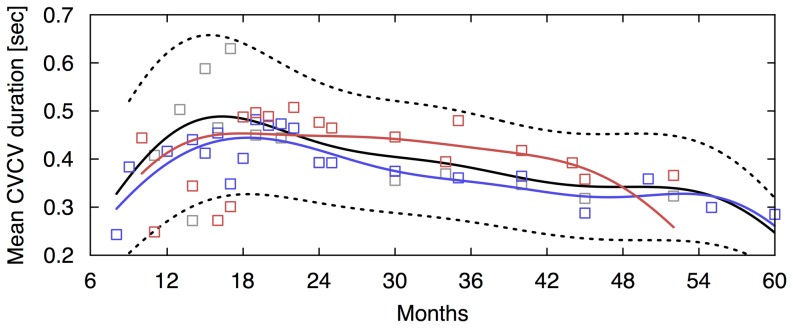
The developmental changes of durations of CVCV sequences in Japanese children’s speech. The solid and dash lines denote mean durations of CVCV sequences and ± 1 standard deviations obtained from the group data. The blue, red and gray markers show the individual analysis for the Japanese children denoting means duration of CVCV sequences.

For the developmental changes in the preference for the direction of articulations (Figure 2ii), although values of the fronting index fluctuated over time, both children showed early preference to the fronting patterns. This preference exists around 12 months and seems to decrease after 18 months (R^2^ = 0.670 and 0.158 for child B and C, respectively). For the development of intra-organ articulations (Figure 2iii), we obtained a developmental curve (R^2^ = 0.833 and 0.278 for child B and C, respectively). For the development of inter-organ articulations (Figure 2iv), we obtained two developmental curves (Figure 2iv), that is, C_L_VC_C_ + C_C_VC_L_ (R^2^ = 0.708 and 0.268 for child B and C, respectively) and C_L_VC_D_ + C_D_VC_L_ (R^2^ = 0.468 and 0.664 for child B and C, respectively). For child B, normalized ratios of intra-articulatory development were initially higher than those of inter-articulatory development (labial and coronal, and labial and dorsal consonants patterns). Yet, once the ratios of each of labial-coronal and labial-dorsal consonants patterns exceeded those of intra-organ articulations at 19.1 and 14.2 months of age, respectively, they rapidly peaked at 27.6 and 25.1 months of age, respectively. Although actual normalized ratios of child B and C differ from each others, based on patterns of the intersectional and peak months for the intra- and inter-organ articulations, the child C showed a similar pattern of changes, that is, each of the inter-organ articulations exceeded intra-organ ones at 15.1 and 21.0 months of age, respectively, and rapidly peaked at 21.1 and 25.3 months of age, respectively. In addition, we also analyzed the developmental changes in speed of CVCV articulation. Both children showed peaks in the mean duration of CVCV around 18 months after which the value gradually decreased (Figure 3).

### Analysis of group data for Japanese

In order to assess the developmental trends of group data for Japanese, we analyzed pooled data across three children. For the development of repetitions, we obtained a developmental curve as shown in Figure 2i (R^2^ = 0.722). As the individual analysis has shown, almost all the CVCs were generated at the same place of articulation until around 12 months of age after which the ratio of repetition gradually decreased until 24 months.

**Figure 4:**
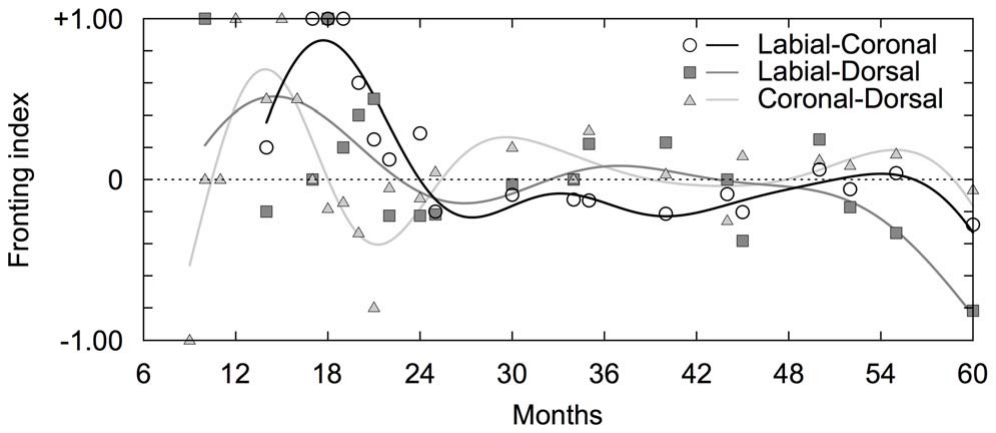
The developmental curve of each fronting pattern in Japanese. The black, gray and silver lines show developmental curves of the labial-vowel-coronal, labial-vowel-dorsal, and coronal-vowel-dorsal sequences, respectively. The circles, squares and triangles show raw fronting indices of the labial-vowel-coronal, labial-vowel-dorsal and coronal-vowel-dorsal sequences, respectively.

For the developmental changes in the preference for the direction of articulations, we obtained a developmental curve of the fronting index as shown in Figure 2ii (R^2^ = 0.200). As a result, we observed that preference to the fronting patterns exists 12 months and peaked around 18 months. This preference gradually decreased over time, and it disappeared after around 24 months. We confirmed that this asymmetry was observed in each of the labial-coronal, labial-dorsal and coronal-dorsal consonants patterns (Figure 4). Although the months at which the curves peaked were different among the three types of sequences, the increasing and decreasing patterns were common.

For the development of intra- and inter-organ articulations, we obtained developmental curves as shown in Figures 2iii and 2iv (R^2^ = 0.752, 0.752, and 0.417 for intra-organ articulations, labial and coronal inter-organ articulations, and labial and coronal inter-organ articulations, respectively). To quantify the developmental trend observed in inter- and intra-organ articulations of Japanese children, we defined durations of development as follows. If a value of the curve exceeded 1/3, we detected this point in time as the onset of developmental change. The point in time at which the value of curve exceeded 2/3 was detected to determine the offset of the developmental period. The durations of developmental change of intra-organ, labial-coronal inter-organ and labial-dorsal inter-organ were 16.5 (from 8.5 to 25.0), 3.3 (from 18.9 to 22.2) and 3.0 (from 16.9 to 19.9) months, respectively. The analysis showed that the intra-articulator temporal relationship was present early on and before the inter-articulator temporal relationship generated speech production. On the other hand, the developmental change of inter-organ articulations was observed to be faster than that of intra-organ articulations.

**Figure 5:**
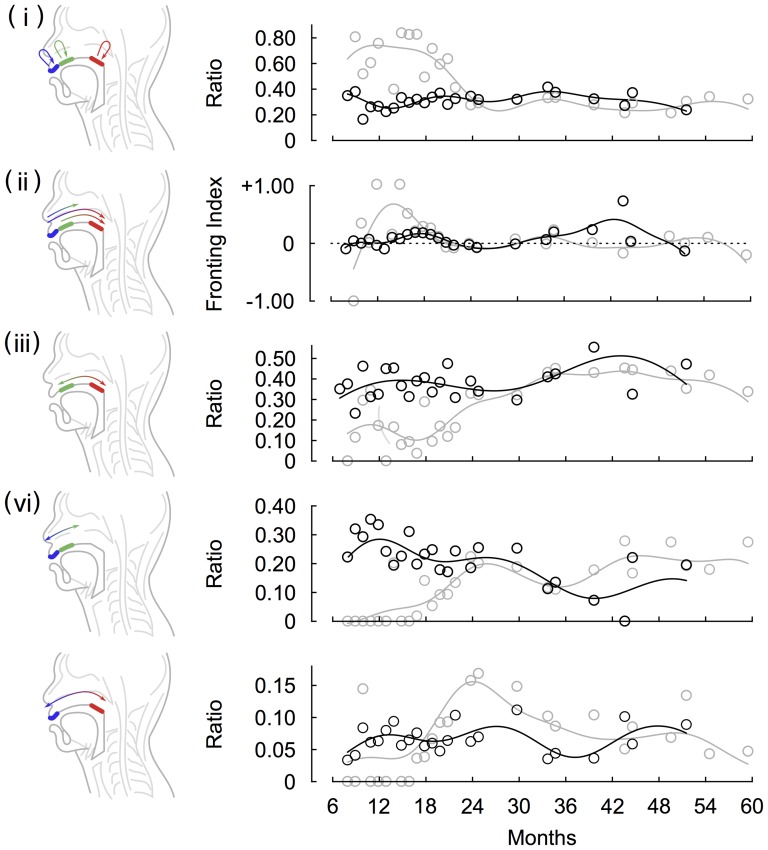
The comparison between change in ratios of the repetitions, fronting, intra- and inter-organ articulations in Japanese child-directed and children’s speech. Changes in the ratio of occurrences of the repetitions, intra- and inter-organ articulations to the total CVC(V) occurrences, and the fronting index were plotted over months of age for Japanese child-directed speech produced by parents (black) and children’s speech (gray).

Regarding the development of speech rate of Japanese, we found non-linear changes, that is, the mean duration of CVCV increased until around 18 months after which the value gradually decreased (Figure 3). In addition, we found that the developmental changes in repetitions, fronting, intra- and inter-organ articulations of Japanese children’s speech were not accompanied by the changes in child-directed speech by their mothers (Figure 5). Yet, the ratios of each type of CVC patterns produced by children tended to converge toward the child-directed speech after 24 months for repetition, fronting, intra-organ articulations and labial-coronal inter-organ articulations and later labial-dorsal inter-organ articulations. Note that, for the child-directed speech, the ratios were globally stable and no preference for the fronting existed.

### Analysis of individual data for English

**Figure 6:**
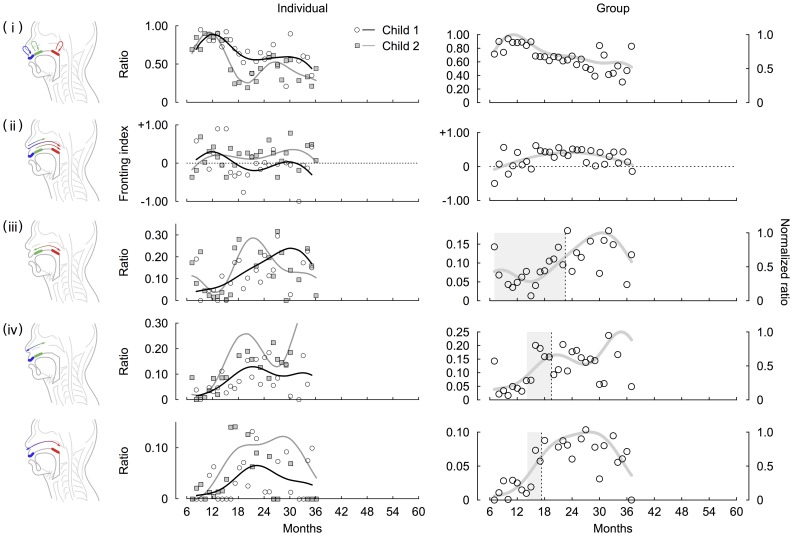
The developmental changes in serial order in articulation of consonants obtained by the analysis of individual and group data for English. The Middle and right columns show developmental curves obtained by the analysis of individual and group data for Japanese, respectively. Each row indicates (i) repetitions, (ii) fronting, (ii) intra-organ articulations, and (iv) inter-organ articulations. In the middle column, circles and squares denote relative ratio of each type of CVC patterns produced by child 1 and child 2, respectively. Black and gray curves indicate that developmental curves of child 1 and child 2, respectively. In the right row, circles and curves denotes that relative ratio of each type of CVC patterns obtained by the analysis of group data and developmental curve of them, respectively. The shaded areas indicate periods between onset and offset of the developmental changes. We defined the onsets and offsets as months at which a value of curves exceeded 1/3 and 2/3, respectively.

We analyzed the individual data for English and obtained developmental curves of the repetition (R^2^ = 0.470 and 0.822 for child 1 and 2, respectively), the fronting tendencies (R^2^ = 0.0841 and 0.0335 for child1 and 2, respectively), the intra-organ articulations (R^2^ = 0.310 and 0.517 for child 1 and 2, respectively), the labial-coronal inter-organ articulations (R^2^ = 0.181 and 0. 741 for child 1 and 2, respectively) and the labial-dorsal inter-organ articulations (R^2^ = 0.200 and 0. 311 for child 1 and 2, respectively). We observed that almost all CVCs were generated by the same place of articulation in early development and that the repetitions decreased from 12 to 24 months (Figure 6i). For the developmental changes in the preference for the direction of articulations, although the values of R^2^ of both children were very low, raw values of the fronting index showed early preferences to fronting patterns for child 1 and 2, and this preference persisted for child 2 (Figure 6ii). For the development of intra- and inter-organ articulations produced, based on the intersectional months of the normalized ratios of both of articulations, both children showed that normalized ratios of intra-articulatory development were initially higher than those of inter-articulatory development (Figures 6iii and 6iv). Yet, once the ratios of the labial-coronal and labial-dorsal inter-organ articulations exceeded those of the intra-organ articulations (For child 1, the labial-coronal and labial-dorsal inter-organ articulation exceeded the intra-organ articulations at 12.1 and 11.1 months, respectively. For child 2, they exceeded the intra-organ articulations at 10.7 and 13.2 months, respectively), they rapidly increased.

### Analysis of group data for English

**Figure 7:**
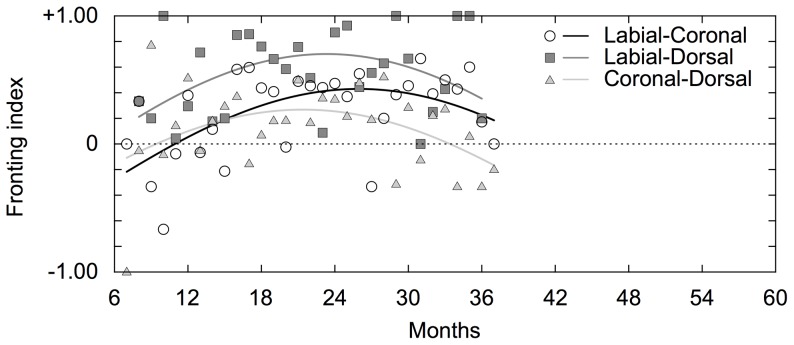
The developmental curve of each fronting pattern in English. The black, gray and silver lines show developmental curves of the labial-vowel-coronal, labial-vowel-dorsal, and coronal-vowel-dorsal sequences, respectively. The circles, squares and triangles show raw fronting indices of the labial-vowel-coronal, labial-vowel-dorsal and coronal-vowel-dorsal sequences, respectively.

We analyzed the group data for English and obtained developmental curves of the repetitions (R^2^ = 0.525), the fronting tendencies (R^2^ = 0.301), the intra-organ articulations (R^2^ = 0.385) and the inter-organ articulations (R^2^ = 0.449 and 0.560 for labial-coronal and labial-dorsal patterns, respectively). The results showed a predominance of repetitions by around 12 months of age and gradually decreased within the range of the tracking periods (Figure 6i). The preferences to the fronting patterns initially increased and decreased at around 24 months (Figure 6ii). Note that, although previous studies reporting fronting tendencies mainly focus on only labial-coronal patterns [11], [12], this asymmetry was also observed in labial-dorsal and coronal-dorsal patterns (Figure 7). While the asymmetries of labial-coronal and labial-dorsal pattern persisted until 36 months, those of coronal-dorsal pattern declined to be around zero (Figure 7). As for the intra- and inter-organ articulations, we calculated durations of these developmental changes of them in the same way as was done for the analysis of Japanese. The durations of developmental change of intra-organ, labial-coronal inter-organ and labial-dorsal inter-organ were 15.6 (from 7.0 to 22.6), 5.9 (from 13.7 to 19.6) and 3.4 (from 14.0 to 17.4) months, respectively. These results also imply later presence and steeper development of the inter-articulatory temporal relationship than the intra-articulatory temporal relationship (Figures 6iii and 6iv).

## Discussion

In the present study, we analyzed longitudinal data of Japanese children and found common developmental trends in CVC sequences among children. Then, we obtained the developmental curves for the group data of Japanese (Figure 2). The onset and duration of the developmental changes were quantified in each curve for the intra- and inter-organ articulations (Figures 2iii and 2iv). In the same way as was done for the analysis of Japanese children, we also conducted analysis for English children (Figure 6). As consequences, although actual ratios differ among children and between both languages, we obtained similar developmental trends in both languages. First, until around 18 months, CVC sequences were dominated by repetitions. After 18 months, these sequences decreased. Second, the group analysis showed that, until 24 months, place of articulation was ordered from those produced at the front of the mouth to those produced in the back. Note that English children prefer the fronting patterns after 24 months. Third, CVC sequences generated by movements within the same articulatory organs were already present around 8 months and then gradually increased. Fourth, sequences generated by movements between different articulatory organs tended to appear later but then rapidly increased after the appearance. Finally, we observed a great change around 18 months in not only the ratio of sequences produced by children but also the duration of these sequences.

### Repetitive articulations

Our results support findings from previous studies [23], [33] that infants tend to repeat articulations at the same place of an organ in the period of babbling (Figures 2i and 6i). Focusing on the neuromuscular coordination, rhythmic mandibular oscillations have been thought to play an important role in speech production during early development [11], [12]. Kinematic studies report that infants have independent control only over their jaw and limited control of upper and lower lips especially at one year of age [20], [34]. Earlier maturation of control over the jaw would be consistent with the mandibular oscillation theory, which are produced by the central pattern generators in the brain stem [12], [19]. From a phonological perspective, it is unlikely that the infants’ linguistic environment cause preferences for repetitions of the same consonants in successive syllables, since preference for variegation of consonants rather than their duplications is generally observed in adult languages [35]. As shown in the Figure 5, this discrepancy between children and adults was also observed in the Japanese child-directed speech contained in the corpus until 24 months. Regarding the preference for variegated sequencing in adult languages, it is argued that variegation would require less energy consumption for the jaw than repetitions: for example, a labial-vowel-coronal sequence (e.g.,/pata/and/tapa/) can be produced by jaw movements with a single cycle, whereas a repetitive sequence (e.g.,/papa/and/tata/) rather requires two jaw cycles [7]. It can be also argued that, since a discrete movement generating a syllable requires an initiation and termination of movements [36], combining two different discrete movements is not always harder than combining the same discrete movements into a sequence. In the present study, we have shown that the ratio of repetitions gradually decreases over time. This result implies that early stability of articulation changes from repetitions to variegations in the developmental process under influence of the linguistic environment.

### Preference for direction of articulations

One of the observed characteristics of consonantal changes is the preference for the direction of articulations, namely fronting vs. backing (Figures 2ii, 4, 6ii and 7). Previous studies have reported that children prefer labial-vowel-coronal sequences over coronal-vowel-labial ones [11], [12]. In the present study, we confirmed that this is indeed the case in children younger than 2 years old for both Japanese and English (Figures 2ii, 4, 6ii and 7). This preferential asymmetry could be attributed to stabilities of phase relationships among movements of the jaw, tongue and lips [7]. This idea originates from investigations about rhythmic movements of limbs, in which, as a rate of movement increases, the coordination pattern of limbs is destabilized and another stable coordination pattern emerges [37]. For the articulatory system, as a speech rate increases, phase relationships among movements of articulators cause the preference of a labial-coronal sequence to a coronal-labial one [7]. This shift is caused by a modification of the coordination between the jaw and constrictors. In these changing processes, labial-to-coronal CVCV disyllables are more favored than coronal-to-labial ones, suggesting that the phase relationship among movements of the jaw, tongue and lips in the former is more stable than the one in the latter. Furthermore, other studies have shown that stop consonantal gestures that occur during fronting of consonant-consonant sequences display more overlap than during backings [38]-[40].

The preference for fronting becomes lower over development after their peaks. As for Japanese, the preference disappears after 24 months (Figures 2ii and 4). This finding is consistent with previous reports that there is no preference for labial-vowel-coronal (LC) in Japanese adult speech production [16], [17]. Together with these studies, our results indicate that Japanese children prefer LC sequences and, over time, they shift their production of these sequences closer to the distribution found in adult speech. A cross-language comparison study of adults found that neuromuscular constraints lead to a universal articulatory bias toward sequences, but that a language-specific perceptual bias emerges from the distributional frequencies found in the native language [17]. Thus, LC sequences have a higher articulatory stability but a lower perceptual stability in Japanese adults. Since infants show LC perceptual bias [41], the developmental change observed in this study implies that speech production is highly constrained by motoric factors in early development and then modulated by the specific language inputs. This adjustment to native languages is also reported in Dutch [23]. Their longitudinal study shows a similar distribution of place of articulation patterns between children’s and adult’s speech. In fact, although the Japanese preferences of all of the fronting sequences seem to disappear after 24 months, regarding English, the preference to the labial-coronal and labial-dorsal sequences persisted within the range of the tracking periods.

### Intra- and Inter-articulatory coordination

The main goal of the present study was an examination of the developmental process that shapes the serial order of speech production in terms of intra- and inter-articulatory coordination. A crucial finding of this study is the early emergence of the intra-articulator temporal relationship that produces variegated sequences of consonants in CVC, indicating that changes in the location of the tongue articulation during speech production start in early development (Figures 2iii, 2iv, 6iii and 6iv). Although the mandibular oscillation hypothesis [11], [12] could explain the early speech production, our finding of the early intra-articulatory coordination requiring independent tongue movements to the jaw suggests that the serial coordination of both the jaw and tongue plays an important role starting in early development [42]. This is further supported by a simulation study showing the role of articulators other than the jaw in a single consonant articulation [43]. Together, these studies imply that the serial coordination of articulators generates a single constrict-release gesture starting in the early developmental stage of speech production. This early presence of the intra-articulator temporal relationship is contrasted with the serial coordination of the inter-articulatory temporal relationships, which emerges later around 18 months for Japanese and 15 months for English. This result implies that serial coordination between the lips and tongue is absent when repetition patterns dominate. Intriguingly, although the onset of the inter-articulator temporal relationship appears later than the intra-articulator one, the developmental change of the inter-articulator relationship is steeper. This observation implies that once children acquire more rapid articulatory movements, stability of the inter-articulator temporal relationship increases.

The analysis of the durations of CVCV sequences revealed that the developmental changes in speech rates are non-linear, that is, the mean duration of CVCV increases until around 18 months, after which the value gradually decreases (Figure 3). In early development, the rhythmic nature of a serial order in speech may be represented as non-segmental articulatory gestures. In other words, the goals of articulatory movements are achieved ambiguously. However, with the development of speech, children generate a clearer serial order by linking together discrete articulatory movements. In the early period of this shift, immature motor control may cause a jerky trajectory. Therefore, the mean duration of CVCV can be comparatively shorter during early development and then gradually increase until around 18 months. Moreover, the development of serial coordination involving inter-articulatory temporal relationships leads to an acceleration of serial order in articulations after around 18 months. Previous studies have reported non-segmental features of speech signals produced by children [44] and articulatory rates that increase with age [45]. These reports are consistent with our findings in the present study. Changes in the functional domain may be related to anatomical modifications of the vocal tract. In fact, other studies have shown that development of the vocal tract shows a non-linear change around 18 months [14].

### Limitations of the present study

In this study, we analyzed individual and group data for both Japanese and English, and obtained developmental curves of repetitions, fronting, intra- and inter-organ articulations (Figure 2 and 6). As a result, we observed some common developmental trends among these articulatory patterns. However, as R^2^ values shows particularly in the analysis of individual data, accuracies of some of the curve fitting were not good. The lower goodness-of-fit might be caused by outliers with a small number of samples. Especially, in early development, unreliability of transcription of ambiguous utterances would also lead to lower accuracies of curve fitting. In this sense, direct measurements or estimations of articulatory movements are needed in future studies.

## Conclusion

In conclusion, our study has shown that the development of serial order in speech undergoes great changes around 18 months for Japanese and 15 months for English. Before these periods, a passive synchronization among the jaw-tongue-lip system, mainly driven by the mandibular oscillation, may generate the repetitions. During the same period, the serial coordination of intra-articulator is present but has limited properties. In order to stabilize articulations, serial coordination during this period has a rhythmic nature. Over time, pressure for the child to produce more informative communication induces the rhythmic movements to differentiate into combinations of discrete movements. Furthermore, increased speed of articulations causes the shift in stability, that is, the serial integration of inter-articulators becomes more stable than that intra-articulator serial integration. Thus, after 18 months for Japanese and 15 months for English, articulations produced by different organs emerge and develop rapidly. On the other hand, the serial coordination of intra-articulators exhibit prolonged development to refine the rapid sequences of articulations. Ultimately, our study shows the manner by which the developing neuromuscular control of articulatory coordination constrains the serial order in speech production among children in both Japanese and English.

## Supporting Information

Table S1 The Number of CVCs in the Japanese corpus [25].
(DOCX)

Table S2 The number of CVCs in child-directed speech in the Japanese corpus [25].
(DOCX)

Table S3 The number of CVCs in the English corpus [29], [30].
(DOCX)

Method S1 **The detail procedures of the kernel regression.** The pseudo-code of the kernel regression used in the present study is as follows. In the pseudo-code, a normal (e.g., *x*), bold symbol (e.g., **X**), superscript symbol ^T^ and the symbol **I** represents a vector, matrix, transpose operator and unit matrix, respectively.
(DOCX)
