# Supplementary material for: Development of a Serial Order in Speech Constrained by Articulatory Coordination
Source: PLoS One. 2013 Nov 5;8(11):e78600. doi: 10.1371/journal.pone.0078600 (PMC3818465; doi:10.1371/journal.pone.0078600)
Supplement: Table S1 — The Number of CVCs in the Japanese corpus [25]. (DOCX) [file pone.0078600.s001.docx]

**Table S1. The Number of CVCs in the Japanese corpus [25]**

| **M.O.** | **Repetitions** | | | | **Intra-Organ** | | | | **Inter-Organ** | | | | | | | | **# Total** |
| --- | --- | --- | --- | --- | --- | --- | --- | --- | --- | --- | --- | --- | --- | --- | --- | --- | --- |
|  |  |  |  |  |  |  |  |  | **Labial-Coronal** | | | | **Labial-Dorsal** | | | |  |
|  | **# Gr.** | **# Individual** | | | **# Gr.** | **# Individual** | | | **#Gr.** | **# Individual** | | | **# Gr.** | **# Individual** | | |  |
| 7 | 0 | 0 | 0 | 0 | 0 | 0 | 0 | 0 | 0 | 0 | 0 | 0 | 0 | 0 | 0 | 0 | 0 |
| 8 | 1 | 1 | 0 | 0 | 0 | 0 | 0 | 0 | 0 | 0 | 0 | 0 | 0 | 0 | 0 | 0 | 1 |
| 9 | 8 | 8 | 0 | 0 | 1 | 1 | 0 | 0 | 0 | 0 | 0 | 0 | 0 | 0 | 0 | 0 | 9 |
| 10 | 4 | 0 | 4 | 0 | 2 | 0 | 2 | 0 | 0 | 0 | 0 | 0 | 1 | 0 | 1 | 0 | 7 |
| 11 | 4 | 0 | 1 | 3 | 2 | 0 | 2 | 0 | 0 | 0 | 0 | 0 | 0 | 0 | 0 | 0 | 6 |
| 12 | 10 | 5 | 4 | 1 | 2 | 0 | 2 | 0 | 0 | 0 | 0 | 0 | 0 | 0 | 0 | 0 | 12 |
| 13 | 1 | 0 | 0 | 1 | 0 | 0 | 0 | 0 | 0 | 0 | 0 | 0 | 0 | 0 | 0 | 0 | 1 |
| 14 | 11 | 9 | 1 | 1 | 4 | 4 | 0 | 0 | 5 | 4 | 0 | 1 | 5 | 5 | 0 | 0 | 25 |
| 15 | 12 | 7 | 0 | 5 | 1 | 1 | 0 | 0 | 0 | 0 | 0 | 0 | 0 | 0 | 0 | 0 | 13 |
| 16 | 40 | 30 | 2 | 8 | 4 | 2 | 2 | 0 | 0 | 0 | 0 | 0 | 0 | 0 | 0 | 0 | 44 |
| 17 | 51 | 17 | 1 | 33 | 2 | 2 | 0 | 0 | 1 | 0 | 1 | 0 | 2 | 2 | 0 | 0 | 56 |
| 18 | 43 | 39 | 4 | . | 22 | 21 | 1 | . | 11 | 10 | 1 | . | 3 | 3 | 0 | . | 79 |
| 19 | 60 | 39 | 4 | 17 | 7 | 4 | 2 | 1 | 4 | 3 | 0 | 1 | 5 | 4 | 0 | 1 | 76 |
| 20 | 72 | 59 | 13 | . | 18 | 10 | 8 | . | 10 | 5 | 5 | . | 10 | 8 | 2 | . | 110 |
| 21 | 61 | 35 | . | 26 | 10 | 10 | . | 0 | 8 | 7 | . | 1 | 8 | 8 | . | 0 | 87 |
| 22 | 55 | 46 | 9 | . | 19 | 9 | 10 | . | 16 | 10 | 6 | . | 31 | 27 | 4 | . | 121 |
| 24 | 182 | 49 | 109 | 24 | 190 | 33 | 141 | 16 | 132 | 26 | 94 | 12 | 93 | 19 | 44 | 30 | 597 |
| 25 | 89 | 42 | 47 | . | 86 | 42 | 44 | . | 55 | 35 | 20 | . | 46 | 29 | 17 | . | 276 |
| 30 | 80 | 23 | 41 | 16 | 70 | 21 | 48 | 1 | 42 | 20 | 22 | 0 | 33 | 18 | 14 | 1 | 225 |
| 34 | 51 | . | 47 | 4 | 58 | . | 53 | 5 | 16 | . | 16 | 0 | 14 | . | 12 | 2 | 139 |
| 35 | 78 | 60 | 18 | . | 92 | 84 | 8 | . | 23 | 20 | 3 | . | 18 | 16 | 2 | . | 211 |
| 40 | 155 | 87 | 17 | 51 | 211 | 118 | 28 | 65 | 89 | 57 | 8 | 24 | 52 | 32 | 2 | 18 | 507 |
| 44 | 19 | . | 19 | . | 35 | . | 35 | . | 22 | . | 22 | . | 4 | . | 4 | . | 80 |
| 45 | 260 | 179 | 3 | 78 | 347 | 239 | 7 | 101 | 133 | 92 | 2 | 39 | 68 | 42 | 0 | 26 | 808 |
| 50 | 28 | 28 | . | . | 50 | 50 | . | . | 32 | 32 | . | . | 8 | 8 | . | . | 118 |
| 52 | 147 | . | 55 | 92 | 149 | . | 78 | 71 | 83 | . | 25 | 58 | 58 | . | 4 | 54 | 437 |
| 55 | 53 | 53 | . | . | 57 | 57 | . | . | 25 | 25 | . | . | 6 | 6 | . | . | 141 |
| 60 | 84 | 84 | . | . | 77 | 77 | . | . | 64 | 64 | . | . | 11 | 11 | . | . | 236 |

Note: Each number indicates the number of occurrences of each type of CVC.

M.O.: month-olds, # Gr.: number of samples in the group data, # Individual: number of samples in the individual children, # Total: number of samples of total CVC(V) sequences
