# Supplementary material for: Development of a Serial Order in Speech Constrained by Articulatory Coordination
Source: PLoS One. 2013 Nov 5;8(11):e78600. doi: 10.1371/journal.pone.0078600 (PMC3818465; doi:10.1371/journal.pone.0078600)
Supplement: Table S2 — The number of CVCs in child-directed speech in the Japanese corpus [25]. (DOCX) [file pone.0078600.s002.docx]

**Table S2. The number of CVCs in child-directed speech in the Japanese corpus [25]**

| **M.O.** | **Repetition** | **Within Organ** | **Different Organs** | | **#Total** |
| --- | --- | --- | --- | --- | --- |
|  |  |  | **Labial-Coronal** | **Labial-Dorsal** |  |
| 7 | 66 | 40 | 7 | 5 | 118 |
| 8 | 35 | 33 | 20 | 3 | 91 |
| 9 | 73 | 39 | 55 | 7 | 174 |
| 10 | 22 | 54 | 35 | 10 | 121 |
| 11 | 43 | 45 | 52 | 9 | 149 |
| 12 | 141 | 151 | 159 | 30 | 481 |
| 13 | 85 | 149 | 82 | 27 | 343 |
| 14 | 87 | 137 | 60 | 29 | 313 |
| 15 | 152 | 146 | 92 | 23 | 413 |
| 16 | 153 | 142 | 144 | 30 | 469 |
| 17 | 217 | 231 | 120 | 46 | 614 |
| 18 | 231 | 280 | 164 | 39 | 714 |
| 19 | 164 | 143 | 108 | 26 | 441 |
| 20 | 226 | 206 | 98 | 26 | 556 |
| 21 | 108 | 160 | 59 | 22 | 349 |
| 22 | 218 | 181 | 146 | 62 | 607 |
| 24 | 222 | 220 | 107 | 36 | 585 |
| 25 | 46 | 43 | 33 | 9 | 131 |
| 30 | 48 | 39 | 34 | 15 | 136 |
| 34 | 66 | 57 | 16 | 5 | 144 |
| 35 | 124 | 123 | 40 | 13 | 300 |
| 40 | 10 | 15 | 2 | 1 | 28 |
| 44 | 3 | 6 | 0 | 1 | 10 |
| 45 | 64 | 49 | 34 | 9 | 156 |
| 52 | 15 | 26 | 11 | 5 | 57 |

Note: The notation is the same as that used in Table S1.
