# Supplementary material for: Development of a Serial Order in Speech Constrained by Articulatory Coordination
Source: PLoS One. 2013 Nov 5;8(11):e78600. doi: 10.1371/journal.pone.0078600 (PMC3818465; doi:10.1371/journal.pone.0078600)
Supplement: Table S3 — The number of CVCs in the English corpus [29], [30]. (DOCX) [file pone.0078600.s003.docx]

**Table S3. The number of CVCs in the English corpus [29,30]**

| **M.O.** | **Repetitions** | | | **Intra-Organ** | | | **Inter-Organ** | | | | | | **# Total** |
| --- | --- | --- | --- | --- | --- | --- | --- | --- | --- | --- | --- | --- | --- |
|  |  |  |  |  |  |  | **Labial-Coronal** | | | **Labial-Dorsal** | | |  |
|  | **# Gr.** | **# Individual** | | **# Gr.** | **# Individual** | | **# Gr.** | **# Individual** | | **# Gr.** | **# Individual** | |  |
| 7 | 10 | . | 8 | 2 | . | 2 | 2 | . | 1 | 0 | . | 0 | 14 |
| 8 | 251 | 19 | 119 | 19 | 5 | 11 | 6 | 1 | 0 | 3 | 0 | 3 | 279 |
| 9 | 131 | 4 | 96 | 35 | 0 | 31 | 6 | 0 | 1 | 5 | 0 | 4 | 177 |
| 10 | 1044 | . | 446 | 48 | . | 22 | 18 | . | 4 | 1 | . | 0 | 1111 |
| 11 | 701 | 52 | 202 | 28 | 3 | 5 | 39 | 3 | 8 | 23 | 3 | 3 | 791 |
| 12 | 598 | 51 | 162 | 33 | 3 | 3 | 29 | 2 | 16 | 17 | 4 | 1 | 677 |
| 13 | 840 | 39 | 321 | 59 | 0 | 6 | 30 | 2 | 7 | 14 | 0 | 5 | 943 |
| 14 | 1449 | 13 | 152 | 134 | 2 | 7 | 122 | 2 | 16 | 17 | 0 | 3 | 1722 |
| 15 | 1165 | 105 | 190 | 17 | 0 | 1 | 94 | 3 | 20 | 25 | 0 | 9 | 1301 |
| 16 | 1507 | 31 | 33 | 89 | 3 | 2 | 441 | 2 | 28 | 161 | 0 | 11 | 2198 |
| 17 | 1489 | 113 | 17 | 168 | 28 | 17 | 417 | 7 | 23 | 126 | 5 | 10 | 2200 |
| 18 | 1222 | 62 | 12 | 144 | 18 | 13 | 288 | 7 | 8 | 159 | 6 | 11 | 1813 |
| 19 | 1003 | 29 | 6 | 171 | 3 | 14 | 257 | 17 | 6 | 196 | 4 | 4 | 1627 |
| 20 | 300 | 26 | . | 49 | 4 | . | 41 | 6 | . | 53 | 1 | . | 443 |
| 21 | 283 | 17 | 12 | 60 | 16 | 11 | 47 | 4 | 5 | 33 | 6 | 2 | 423 |
| 22 | 232 | 27 | 12 | 36 | 7 | 7 | 77 | 8 | 19 | 33 | 6 | 4 | 378 |
| 23 | 442 | 54 | 14 | 131 | 7 | 7 | 75 | 5 | 4 | 57 | 13 | 5 | 705 |
| 24 | 352 | 41 | . | 40 | 15 | . | 91 | 12 | . | 31 | 1 | . | 514 |
| 25 | 224 | 45 | 21 | 51 | 8 | 7 | 73 | 13 | 3 | 53 | 1 | 3 | 401 |
| 26 | 128 | 27 | 11 | 23 | 5 | 2 | 31 | 2 | 4 | 18 | 0 | 0 | 200 |
| 27 | 90 | 47 | 9 | 42 | 28 | 6 | 24 | 8 | 3 | 18 | 7 | 0 | 174 |
| 28 | 65 | . | 4 | 21 | . | 3 | 20 | . | 2 | 27 | . | 4 | 133 |
| 29 | 35 | 9 | 4 | 35 | 22 | 0 | 13 | 6 | 1 | 7 | 4 | 2 | 90 |
| 30 | 162 | 134 | 24 | 14 | 4 | 6 | 11 | 2 | 8 | 6 | 2 | 3 | 193 |
| 31 | 70 | . | . | 16 | . | . | 6 | . | . | 8 | . | . | 100 |
| 32 | 40 | 4 | 3 | 18 | 3 | 1 | 23 | 0 | 3 | 16 | 0 | 3 | 97 |
| 33 | 32 | 14 | . | 11 | 4 | . | 24 | 4 | . | 7 | 0 | . | 74 |
| 34 | 68 | 39 | 7 | 30 | 15 | 5 | 21 | 4 | 8 | 7 | 5 | 0 | 126 |
| 35 | 20 | 7 | 4 | 17 | 3 | 3 | 25 | 7 | 11 | 4 | 2 | 0 | 66 |
| 36 | 33 | . | 20 | 3 | . | 1 | 29 | . | 22 | 5 | . | 0 | 70 |
| 37 | 34 | . | . | 5 | . | . | 2 | . | . | 0 | . | . | 41 |

Note: The notation is the same as that used in Table S1.
