# Supplementary material for: Development of a Serial Order in Speech Constrained by Articulatory Coordination
Source: PLoS One. 2013 Nov 5;8(11):e78600. doi: 10.1371/journal.pone.0078600 (PMC3818465; doi:10.1371/journal.pone.0078600)
Supplement: Method S1 — The detail procedures of the kernel regression. The pseudo-code of the kernel regression used in the present study is as follows. In the pseudo-code, a normal (e.g., x), bold symbol (e.g., X), superscript symbol T and the symbol I represents a vector, matrix, transpose operator and unit matrix, respectively. (DOCX) [file pone.0078600.s004.docx]

**Method S1. The detail procedures of the kernel regression**

The pseudo-code of the kernel regression used in the present study is as follows. In the pseudo-code, a normal (e.g., *x*), bold symbol (e.g., **X**), superscript symbol ^T^ and the symbol **I** represents a vector, matrix, transpose operator and unit matrix, respectively.

1: Input: **X**, **T**, *N* and *σ* denote independent variables, predictors, the number of samples and the parameter of radial basic function (RBF) kernel, respectively. We decided *σ* by the leave-one-out cross-validation.

2: We calculate the Gramian matrix $\boldsymbol{\phi}$ using RBF kernel by following equations: **𝛟←{**𝜙(𝑥𝑖,**𝐗**)}, where ${\phi(x}_{i},\mathbf{X})=exp(-{(x_{i}-\mathbf{X})}^{2}/\sigma)$*.*

3: $\alpha, \beta\leftarrow\mathrm{random}\text{ number}\text{s}$

Our aim is to obtain optimal weights coefficient $\mathbf{w}$ of the Gramian matrix $\boldsymbol{\phi}$. We assumed the hyper-parameters $\alpha$, which determines variance of weight coefficients, and $\beta$, which determines variance of noise. In order to obtain optimal $\mathbf{w}, \alpha$ and $\beta$, we conduct an iterative optimization. Initially, we set random values to $\alpha$ and $\beta$, and then conducted the iterative optimization.

4: **loop**

5: By using given $\alpha$ and $\beta$, posterior distribution of **w** is given by the following equation: $p\left( \mathbf{w},\mathbf{T} \right)=N(\mathbf{w}|\mathbf{M},\mathbf{S})$. We can calculate the mean **M** and variance **S** of the distribution by the following equations.

$\mathbf{S}^{\boldsymbol{-}\boldsymbol{1}}\leftarrow\alpha\mathbf{I}+\beta\boldsymbol{\phi}^{T}\boldsymbol{\phi}$,

$$\mathbf{M}\boldsymbol{\leftarrow}\beta\mathbf{S}\boldsymbol{\phi}^{T}\mathbf{T}$$

6: We update $\alpha$ and $\beta$ under given posterior distribution of **w** by maximizing marginal likelihood function $p\left( \mathbf{T} | \alpha,\beta\right)=\int p\left( \mathbf{T} | \mathbf{w},\beta\right)p\left( \mathbf{w} | \alpha\right)d\mathbf{w}$. First, we update $\alpha$ by the following equations:

$\alpha\leftarrow\gamma/\mathbf{M}^{T}\mathbf{M}$, and

$\gamma\leftarrow\sum\lambda/ (\alpha+\lambda)$, where $\lambda$ is eigen vector of $\beta\boldsymbol{\phi}^{T}\boldsymbol{\phi}$**.**

7: Next, we update $\beta$ by the following equation.

$$\beta\leftarrow\sum\left( \mathbf{T}-\boldsymbol{\phi M} \right)^{2}/\left( N-\gamma\right)$$

11: **end loop**

12: We conduct the above loop 100 times and finally obtained the predictive variables by calculating $\boldsymbol{\phi M}$**.**
